# Case report: The effect of second-line vebreltinib treatment on a patient with advanced NSCLC harboring the MET exon 14 skipping mutation after tepotinib treatment

**DOI:** 10.3389/fonc.2024.1331387

**Published:** 2024-04-19

**Authors:** Siyuan Huang, Linlin Li, Ningning Yan, Huixian Zhang, Qianqian Guo, Sanxing Guo, Di Geng, Xincheng Liu, Xingya Li

**Affiliations:** ^1^ Oncology Department, The First Affiliated Hospital of Zhengzhou University, Zhengzhou, China; ^2^ Department of Medicine, Beijing Pearl Biotechnology Co., Ltd, Beijing, China

**Keywords:** vebreltinib, case report, METex14 skipping mutation, non-small cell lung cancer, treatment resistance

## Abstract

**Background:**

Highly selective type Ib mesenchymal–epithelial transition gene (MET) tyrosine kinase inhibitors (TKIs) are the standard-of-care (SOC) therapy for previously untreated non-small cell lung cancer (NSCLC) harboring MET exon 14 (METex14) skipping mutations. However, there are rare reports describing effective regimens for patients who fail SOC without identifying resistant mutations or tissue transformation.

**Case report:**

We report the first case of a 74-year-old woman with lung adenocarcinoma (cT1cNxM0) harboring METex14 splice region mutation, which was identified by a next-generation sequencing (NGS)-based assay. The patient was administered two treatments, including first-line tepotinib and second-line vebreltinib. The patient achieved progression-free survival (PFS) of 7.6 months, and then disease progression of tepotinib was observed. A re-biopsy was performed for NGS, which revealed the same mutations as before, with no new gene mutations detected. The woman received subsequent vebreltinib therapy and experienced durable clinical benefits. In the first 6.8 months, chest computed tomography demonstrated stable disease. Then, she achieved partial response (PR). The durable PR lasted for more than 13 months, and the PFS is currently over 20 months, exceeding the prior treatment.

**Conclusion:**

This case highlights the importance of considering re-biopsy and reanalysis of genetic profiles in NSCLC patients harboring METex14 skipping mutations after progressive disease in MET TKI treatment. This raises the possibility that vebreltinib may have long-term survival benefits for patients without mutations conferring resistance (funded by Beijing Pearl Biotechnology Co., Ltd; ClinicalTrials.gov number, NCT04258033).

## 1 Introduction

Mesenchymal–epithelial transition gene (MET) alterations have been identified as an oncogenic driver in non-small cell lung cancer (NSCLC) (1). MET exon 14 skipping (METex14) mutations are splice-site oncogenic mutations found in approximately 2%–3% of NSCLC patients. These mutations are associated with tumor proliferation, invasion, and metastasis (2). Patients with such characteristics respond well to MET tyrosine kinase inhibitors (TKIs). The National Comprehensive Cancer Network (NCCN) guidelines and the American Society of Clinical Oncology (ASCO) guidelines preferentially recommend MET TKIs as the preferred therapy for patients with sensitive mutations who have not received MET TKI treatment (3, 4). Selective MET inhibitors, currently available worldwide as a first-line treatment, have shown a median progression-free survival (PFS) from 6 to 13 months for patients treated with them. Data from patients treated with selective MET TKIs in second- or later-line therapy were analyzed. Median PFS was from 5.5 to 11 months (1). However, there is a rare report of patients treated with MET TKIs after progression. Historically, patients with positive driver genes have limited benefit from immunotherapy or chemotherapy (5). Therefore, optimal treatment strategies for these patients need to be explored.

Vebreltinib (PLB1001) is a highly selective type Ib MET TKI, similar to tepotinib and capmatinib, which blocks ATP binding sites to prevent phosphorylation avoiding activation of the MET receptor. It displayed a generally well-tolerated safety profile and preliminary evidence of clinical activity in patients with NSCLC harboring different MET alterations (6, 7). Vebreltinib also showed a promising clinical response in patients with NSCLC harboring METex14 skipping mutation in KUNPENG, a phase II trial, which reported an objective response rate of 75% (95% confidence interval [CI], 61.1∼86.0) and a median duration of response (DoR) of 15.9 months (95% CI, 9.2∼17.8) per blinded independent review committee (7). In addition, it could assist patients with secondary glioblastoma harboring PTPRZ1-MET fusion in achieving a partial response (PR) with detectable drug concentration in cerebrospinal fluid in a clinical trial (NCT06105619) (8). This may potentially aid in reducing the risk of NSCLC with brain metastases potentially.

To the best of our knowledge, this is the first report of a patient with such an outcome. Herein, the case for the lung adenocarcinoma harboring the METex14 skipping mutation and treated with the second-line vebreltinib in our hospital is as follows.

## 2 Case report

**Figure 1:**
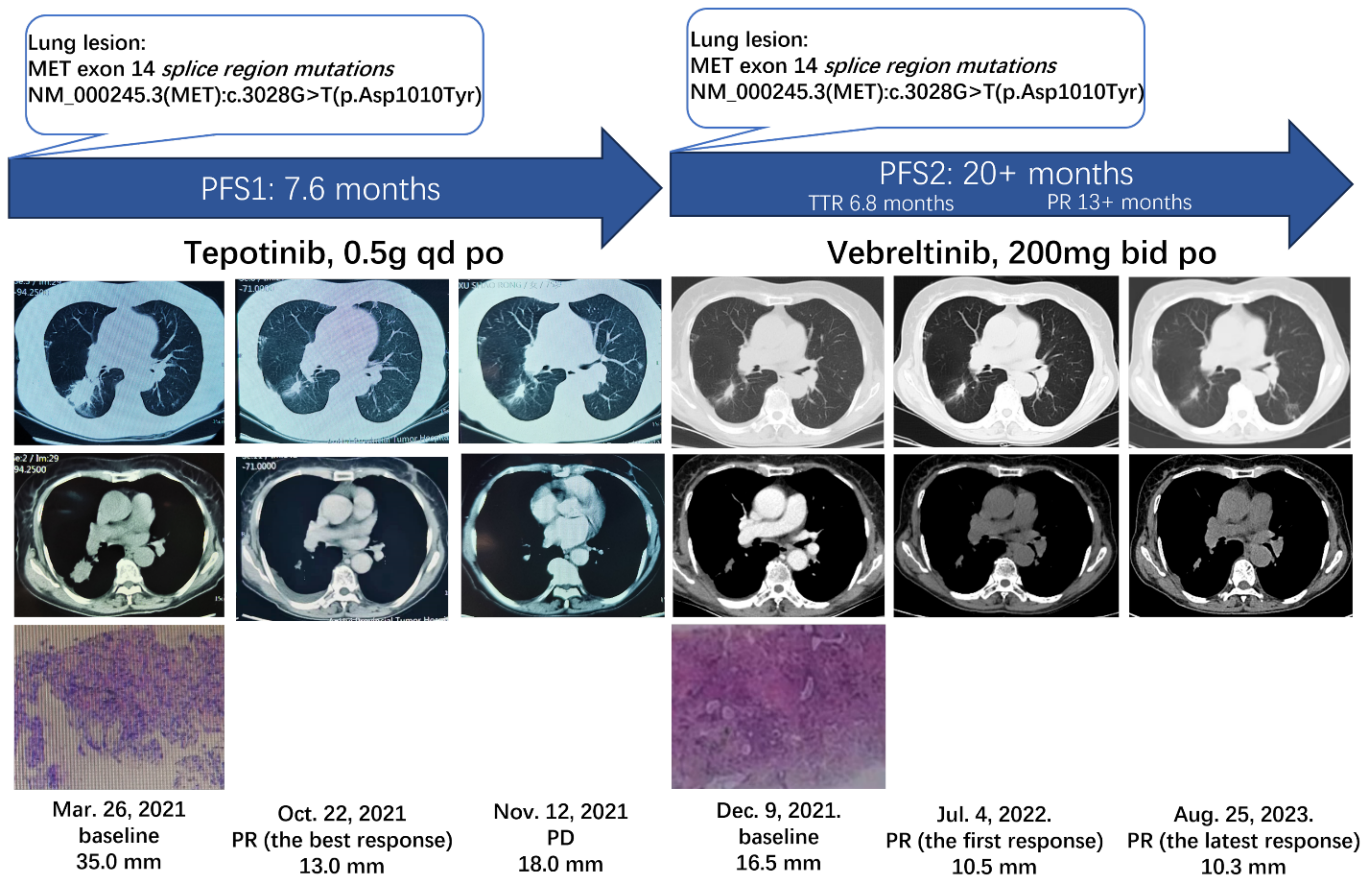
Clinical and treatment course of the patient. MET, mesenchymal–epithelial transition gene; PFS, progression-free survival; TTR, time to response; PR, partial response; po, per OS; PD, progressive disease.

A 74-year-old non-smoking woman was diagnosed with the right lung shadow following a chest computed tomography (CT) scan (**Figure 1**). She was admitted to the hospital because of coma after misusing hypnotics. Primary lung adenocarcinoma was diagnosed through radiological examinations and a lung biopsy in the right lower lobe. Immunohistochemically, it showed TTF-1 (+), Napsin A (+), CK5/6 (−), P40 (−), Ki-67 (<5%+), and AE1/AE3 (+). Surgery was not pursued due to concerns regarding age and tolerability. Enhanced CT scan revealed a 35 mm * 25 mm mass in the dorsal segment of the lower lobe in the right lung, accompanied by obstructive inflammation. Multiple slightly enlarged lymph nodes were observed in the mediastinum and hilum of the right lung (cT2N2Mx). At that time, the formalin-fixed paraffin-embedded (FFPE) tissue was examined using an NGS-based assay (Covance CLS China) and the METex14 splice region mutation was identified as c.3028G>T with the abundance of 18.81%. The woman began orally administering tepotinib (Merck-0.5g, once daily). After 1 month, a CT scan revealed that the mass on the right lung lesion had decreased in size to 14 mm * 13 mm. After nearly 5 months since the discontinuation of tepotinib, a chest CT revealed that the mass had reduced to 13 mm * 11 mm, marking the best response. However, the lesion in the right lung increased to 18 mm * 12 mm after 7.6 months of tepotinib treatment, indicating disease progression. Afterward, tepotinib treatment was discontinued.

Additionally, at this time, radiological examinations confirmed the diagnosis of NSCLC, with enlarged mediastinal, right hilar, and bilateral inguinal lymph nodes (cT2N3M1a, stage IVA). In addition, a fine-needle aspiration re-biopsy of the lung was performed for the NGS-based assay (OncoScreen® Focus panel, Burning Rock Dx), which revealed the same METex14 splice region mutations as previously identified, NM_000245.3(MET): c.3028G>T(p. Asp1010Tyr), without any newly identified resistant mutations. The patient was then enrolled in cohort 4 of a clinical trial (KUNPENG, NCT04258033) and initiated on vebreltinib (200 mg twice daily) as a second-line therapy. The patient had stable disease for 6.8 months.

The woman achieved her first PR after 6.8 months from vebreltinib treatment, and further reduction was observed in the size of the mass in the right lung (10.5 mm). The most recent chest CT shows an overall decrease (10.3 mm) in the size of the mass in the right lower lobe, and the treatment was ongoing for over 20 months. The DoR and PFS exceeded 13 months and 20 months, respectively. As of the latest visit, she maintained the response without any Grade 3 or above adverse events (AEs) and serious adverse events. No treatment-emergent adverse events led to permanent treatment discontinuation or dose reduction.

The woman developed Grade 2 bilateral lower-extremity edema, which was managed with spironolactone tablets. The patient had mild peripheral edema before starting vebreltinib treatment. A grade 2 AE of anemia was observed twice and recovered after intervention with traditional Chinese medicine. Grade 1 AEs of hyponatremia, amylase increase, and lipase increase were also observed once each, and they recovered without any intervention. A Grade 2 AE of ankle pain was still ongoing but was not considered related to the treatment.

## 3 Discussion

We herein report a rare case of a patient with lung adenocarcinoma harboring non-resistant METex14 mutation who failed in prior tepotinib treatment and did not experience longer DoR and PFS compared with subsequent vebreltinib treatment. This outcome is challenging to explain. There are three generally accepted possible hypotheses. The emergence of the first, new resistant mutations or MET abnormalities emerge due to the “pressure selection” of tepotinib; the second involves other resistance mutations or factors that bypass tepotinib. The potential mechanisms of drug resistance are not clear, and they will be further explored in the future. We suspect that some special drug-resistant mutations beyond the detection range might have occurred. The third is histological transformation (9). We considered that the pharmacokinetics of different drugs and individuals could not be ruled out, resulting in variations in drug exposure. These differences might impact the duration of the patient’s response to the therapeutic drugs. However, this hypothesis needs to be confirmed by pharmacokinetic studies. In addition, a biopsy of a single tumor lesion or metastasis may not reflect the heterogeneous genotype of the tumor and its metastases (10), and all biopsy-based tests are subject to the errors and limitations of invasive tissue collection (11) which might possibly occur in this case.

Vebreltinib and tepotinib are both selective, type Ib c-Met inhibitors, but there is subtle difference in the kinase selectivity profile between them. Vebreltinib is highly selective against a panel of 100 kinases (8, 12), whereas tepotinib showed approximately a 50% inhibition rate for IRAK1 and Mer and stronger inhibition against IRAK4, TrkA, and Axl at the same concentration of 2 μM (13).

Precision-targeted therapy and therapeutic regimens of different lines settings have been continuously investigated for NSCLC patients harboring driver gene mutations (5, 14). Although we reached varying conclusions from different clinical trials, it is still challenging to pinpoint a single regimen that is suitable for all patients. In this case, re-biopsy and reanalysis of genetic profiles in patients with NSCLC should be considered when the first line of targeted therapy results in disease progression. This approach could potentially lead to more personalized precision treatment for these patients. Accordingly, targeted therapy with another highly selective MET TKI may result in greater survival benefits.

## 4 Conclusion

This case report presents a prolonged response duration of vebreltinib in a patient with NSCLC harboring a METex14 skipping mutation who was resistant to tepotinib. We suggest that after the failure of first-line treatment of MET TKI, it is important to consider re-biopsy and reanalysis of the genetic profile of patients with NSCLC. This raises the possibility that vebreltinib may have long-term survival benefits for patients without resistance mutations or tissue transformation.

## Data availability statement

The original contributions presented in the study are included in the article/supplementary material. Further inquiries can be directed to the corresponding author.

## Ethics statement

The studies involving humans were approved by the First Affiliated Hospital of Zhengzhou University. The studies were conducted in accordance with the local legislation and institutional requirements. The participants provided their written informed consent to participate in this study. Written informed consent was obtained from the individual(s) for the publication of any potentially identifiable images or data included in this article. Written informed consent was obtained from the participant/patient(s) for the publication of this case report.

## Author contributions

SH: Writing – original draft. LL: Writing – review & editing. NY: Writing – review & editing. HZ: Writing – review & editing. QG: Writing – review & editing. SG: Writing – review & editing. DG: Writing – review & editing. XCL: Writing – original draft, Writing – review & editing. XYL: Supervision, Writing – review & editing.
